# Effect of oral mifepristone and vaginal misoprostol for early abortion among Indian women

**DOI:** 10.6026/9732063002001363

**Published:** 2024-10-31

**Authors:** Bolde Aishwarya Ashok, Rajkumar P Patange, Supriya Patil

**Affiliations:** 1 Department of Obstetrics and Gynecology, Krishna Institute of Medical Sciences, Karad - 415110, Maharashtra, India

**Keywords:** Surgical, oral, lab investigations, questionnaire, clinically

## Abstract

Surgical and medicinal alternatives are accepted for early abortion. However, their clinical effectiveness, costs and patient experiences differ to determine a preferable method. Therefore, it is of interest to evaluate the oral and surgical procedure for mifepristone and vaginal misoprostol in early abortion. Hence, 150 patients were divided equally for surgical and oral methods to evaluate on the basis of questionnaire and clinical investigations. Data shows that post-operative outcome for women in both the groups showed non- significant difference among them requiring further longitudinal studies.

## Background:

Studies have shown that pregnancy termination and complications associated with abortion continue to be one of the possible causes of maternal mortality and morbidity, posing risks to women's physical and mental health as well as a social and financial burden on communities and health systems [1, 2]. Studies have also shown that approximately 60% of unintended pregnancies result in a deliberate termination, with an estimated 73 million procedures performed each year, indicating a 30% rise since 1999 [3]. A study showed that, around 45% of all abortions take place in unsafe environments, with a staggering 97% occurring in underdeveloped countries. Unsafe abortions are most prevalent in Asia, particularly in South and Central Asia [4]. In another study, it was found that, there may be some inaccuracies in the classification of pregnancy related maternal deaths, but it is worth noting that abortion and its consequences account for 2.3% of maternal fatalities in Iran [5]. Ensuring access to timely, affordable and compassionate abortion care is a pressing issue that impacts both public health and human rights [2]. Medical abortion using prostaglandins, such as mifepristone alone, mifepristone with prostaglandins, and methotrexate with prostaglandins, has grown in popularity in recent years as a non-invasive alternative and it plays an important role in ensuring that women have access to safe, effective and acceptable abortion and post-abortion care [6]. Mifepristone, an antagonist of progesterone, enhances the synthesis of prostaglandins in the uterine endometrium during pregnancy [7]. Additionally, it enhances the contractile capacity of the uterine muscles, increases the sensitivity of the pregnant uterus to externally sourced prostaglandins and induces the softening and dilation of the cervix [7]. For additional purposes, such as inducing labour in the case of fetal death after the first trimester and medically terminating a vital pregnancy, researches have shown that the sequential combination of mifepristone and misoprostol is more effective than using misoprostol alone [8, 9]. Based on the available evidence, it can be concluded that the combination of mifepristone with misoprostol is more effective than using misoprostol alone for treating non-vital pregnancies in the first trimester [9]. Therefore, it is of interest to report oral mifepristone and vaginal misoprostol versus surgical in early abortion among Indian women.

## Materials and Methods:

The current longitudinal prospective observational study was conducted with 150 patients *i.e.* 75 cases undergoing medical termination and 75 cases undergoing surgical abortion in the Obstetrics Department of Krishna Hospital, Karad, a tertiary care center from June 2022 to November 2023, covering 18 months with the help of questionnaires which includes, age, BMI, gravidity, gestational age, history of abortion and side effects. Clinical assessments includes physical examination to assess overall health status, ultrasound to confirm gestational age and exclude contraindications such as ectopic pregnancy and evaluation of vital signs and pre-existing conditions and lab investigations includes CBC (main focus on hemoglobin levels pre- and post-abortion), urine pregnancy test to confirm pregnancy & blood type and Rh factor.

## Inclusion criteria:

[1] Women with gestational age <12 weeks confirmed by ultrasound.

[2] Cases of missed abortion, incomplete abortion, or inevitable abortion.

## Exclusion criteria:

Presence of uterine fibroid, polyp, ectopic pregnancy, pelvic infection or bleeding disorder 

## Statistical analysis:

An analysis of the data was performed using the SPSS program. In order to provide a concise summary of demographic and clinical data, descriptive statistics were used. To compare categorical data, chi-square tests were used, whereas t-tests were utilized to evaluate continuous variables. Statistical significance was established at a level of P < 0.05.

## Results:

Table 1 shows that majority of cases were from aged 20-30 years, with 32.0% to 38.7% of cases falling within this range in both medical and surgical group. There was no significant difference between medical and surgical cases across age groups as the P value was 0.9829. Table 2 shows that, out of 75 cases in both the study groups, majority of cases were belonged to 18.5 to 24.99 kg/m^2^. Out of 75 patients in the medical group, 29 (38.7%) had a BMI between 23 and 24.99 kg/m^2^ and 18 (24%) had a BMI below 18.5 kg/m^2^. In surgical group it was 31 (41.3%) and 19 (25.3%) respectively. Thus, there was no statistically significant difference observed between mean BMI and cases of medical and surgical group as the P value was 0.9843. Table 3 Shows that, gestational ages of 9-10 weeks and 11-12 weeks encompassed the majority of cases in both the groups, accounting for 32 (42.7%) in medical group and 34 (45.3%) in surgical group respectively. Gestational ages of 7-8 weeks constituted a smaller proportion of cases for both medical 14 (18.7%) and surgical 10 (13.3%) group. Thus, there no significant difference between gestational age and cases of medical and surgical group as the p value was 0.5829. Table 4 shows that, out of 75 cases in both the groups, multigravida women accounted for the majority of cases in both medical 46 (61.3%) and surgical 49 (65.3%) group, compared to primigravida women who constituted 29 (38.7%) and 26 (34.7%) respectively. Thus, there is no significance difference found between gravida and the study groups as the p value was 0.6112. Table 5 shows that, out of 75 cases in both the groups, cases without a history of abortion constituted the majority of cases for both medical 54 (72.0%) and surgical 56 (74.7%) interventions, compared to those with a history of abortion, who accounted for 21 (28.0%) and 19 (25.3%) respectively. Thus showed no significant difference between history of abortion and cases of medical and surgical group as the p value was 0.7119. Table 6 shows that, the mean ± standard deviation (SD) of Misoprostol doses administered was 5.03 ± 2.01 and the induction-abortion time in hours was 19.72 ± 7.11. Additionally, the table indicates the percentage of cases requiring evacuation, which accounts for 9 (12%) of the total cases. Table 7 shows that, at baseline, the mean Hb level was 11.7 g/dl (± 2.4) for medical cases and 11.9 g/dl (± 2.1) for surgical cases. After abortion, the mean Hb level decreased to 10.6 g/dl (± 1.9) for medical cases and 11.1 g/dl (± 2.5) for surgical cases. It means that the mean change in Hb level was -1.1 g/dl (± 0.6) for medical cases and -0.8 g/dl (± 0.7) for surgical cases. There is significant reduction in Hb levels among medical abortion compared to surgical cases after abortion as the p value was 0.00054. Table 8 shows that, number of cases with high reduction in Hb (1-2 and >2g/dl) were more in medical abortion group compared to surgical group. P value is less than 0.05; it means that the higher reduction in Hb level is significantly more medical group compare to surgical group. (P =<0.001). Table 9 shows that, medical cases experienced bleeding in all instances 75 (100%), significantly higher than surgical cases 62 (82.7%). (P= <0.001). Medical cases also reported higher rates of cramping (58.7% vs. 33.3%, P < 0.001), nausea (25.3% vs. 8.0%, P = 0.002), and vomiting (16.0% vs. 2.7%, P = 0.005) compared to surgical cases. Fever incidence showed no significant difference between medical 3 (4.0%) and surgical 4 (5.3%) cases (P = 0.698). Table 10 shows that, surgical cases experienced infection in 2 (2.7%) of instances, while medical cases reported no infections. Severe bleeding was observed in 6 (8.0%) of medical cases and 2 (2.7%) of surgical cases, though this difference was not statistically significant (P = 0.1462). Surgical cases required readmission after discharge in 1 (1.3%) of cases, whereas medical cases did not. Medical cases required analgesia more frequently 42 (56.0%) compared to surgical cases 14 (18.7%), demonstrating a statistically significant difference. (P= <0.001) Table 11 shows that, the majority of women expressed satisfaction with their method of treatment, with 71 (94.67%) of medical cases and 63 (84.0%) of surgical cases reporting satisfaction (P = 0.0343). Regarding preference for the same method if needed again, 45 (60.0%) of medical cases and 51 (68.0%) of surgical cases indicated a preference (P = 0.3094), showing no statistically significant difference between the two groups in this regard. Table 12 shows that, for medical abortion, it specifies the cost of two medications: a single tablet of Mifepristone costs Rs. 400 and four tablets of Misoprostol (each costing Rs. 20) amount to Rs. 80. The total cost for medical abortion is therefore Rs. 480. In contrast, the cost for a surgical abortion, specifically D & E, is listed as Rs. 1750. This comparison highlights the significant difference in cost between medical and surgical abortion methods.

## Discussion:

Up to 80% of clinical spontaneous abortions occur before 12 weeks of gestation, affecting 8-20% of pregnancies [10]. Throughout history, the treatment of choice for spontaneous abortion has been uterine evacuation through aspiration curettage. Misoprostol is a viable alternative to this traditional surgical approach, according to numerous studies that have suggested this [11, 12]. In a study conducted by Zhang *et al.* in 2005, it was found that misoprostol can be considered as an alternative to surgical surgery for the treatment of early pregnancy loss. The research demonstrated that misoprostol is effective, safe, well-received by patients and has minimal side effects. Various studies have demonstrated the effectiveness and safety of medicinal therapy for early abortion when compared to the conventional surgical method in certain situations. Based on the data, protocol modifications have been made over time. Currently, the preferred choice for managing early gestational loss in most Spanish institutions is medical therapy with misoprostol [13]. Medical treatment is a secure and controllable approach, with patient satisfaction at least equal to that reported after surgical treatment, according to the findings of multiple studies that compared the safety, tolerability and acceptability of medical abortion therapy to surgical abortion therapy. These studies revealed that medical treatment is more acceptable than surgical abortion therapy [14, 15]. In present study gestational ages of 9-10 weeks and 11-12 weeks encompassed the majority of cases in both the groups, accounting for 32 (42.7%) in medical group and 34 (45.3%) in surgical group respectively. Gestational ages of 7-8 weeks constituted a smaller proportion of cases for both medical 14 (18.7%) and surgical 10 (13.3%) group. The P value (0.5829) suggests no significant difference observed between gestational age and cases of medical and surgical group. Moreover, out of 75 cases in both the groups, cases without a history of abortion constituted the majority of cases for both medical 54 (72.0%) and surgical 56 (74.7%) interventions, compared to those with a history of abortion, who accounted for 21 (28.0%) and 19 (25.3%) respectively. Statistical analysis using the P value (0.7119) indicates no significant difference between history of abortion and cases of medical and surgical group. Furthermore, at baseline, the mean Hb level was 11.7 g/dl (± 2.4) for medical cases and 11.9 g/dl (± 2.1) for surgical cases. After abortion, the mean Hb level decreased to 10.7 g/dl (± 1.9) for medical cases and 11.1 g/dl (± 2.5) for surgical cases. It means that the mean change in Hb level was -1.0 g/dl (± 0.6) for medical cases and -0.8 g/dl (± 0.7) for surgical cases. Statistical analysis using the P values 0.0054 for mean change indicates that there is significant reduction in Hb levels among medical abortion compared to surgical cases after abortion. In a study conducted by Barghazan *et al.* (2023), it was discovered that in the surgical group, 13.1% of participants had a history of previous abortion. Interestingly, in the medical group, this percentage was even higher at 29.7%. The observed difference of 16.6 percentage points is statistically significant (p=0.009), indicating a significant variation in the prevalence of previous abortion between the two groups [16]. In Shuaib & Alharazi (2013) they used misoprostol alone [17] as it has been shown that adding mifepristone had no benefit over misoprostol as an initial treatment [18]. Furthermore, it has been seen that repeated intravaginal doses are more successful than a single oral approach [19]. Moreover, vaginal administration of misoprostol seems to be linked to decreased medication adverse effects [20]. In present study majority of women expressed satisfaction with their method of treatment, with 71 (94.67%) of medical cases and 63 (84.0%) of surgical cases reporting satisfaction (P = 0.0343). Regarding preference for the same method if needed again, 45 (60.0%) of medical cases and 51 (68.0%) of surgical cases indicated a preference (P = 0.3094), showing no statistically significant difference between the two groups in this regard. Moreover, medical abortion, it specifies the cost of two medications: a single tablet of Mifepristone costs Rs. 400 and four tablets of Misoprostol (each costing Rs.20) amount to Rs.80. The total cost for medical abortion is therefore Rs.480. In contrast, the cost for a surgical abortion, specifically Dilation and Evacuation (D & E), is listed as Rs.1750. This comparison highlights the significant difference in cost between medical and surgical abortion methods. Nava *et al.* [21] found that medical therapy had over 80% success rates, minor side effects that could be handled with extra medication and a high degree of patient satisfaction. In Spain, the overall cost of misoprostol-assisted medical care is much lower per patient. Lince *et al.* [22] provided supporting evidence for the extension of medical pregnancy termination in South Africa, in combination with the present manual hoover aspiration services. In low-resource settings, a separate randomized-based cost analysis study concluded that medical therapy for incomplete miscarriages is a more cost-effective approach with higher client acceptance and satisfaction than surgical treatment. The administration of misoprostol at a dosage of 0.8 mg, either vaginally or orally, following pre-treatment with mifepristone at 200 mg, is an effective and secure method for medical abortion within a gestational period of up to 63 days. Consequently, the findings indicated that there were no observed differences in efficacy or duration of bleeding with the addition of oral misoprostol for one week following abortion [23]. Out of the 12,829 women who were eligible for evaluation, 2536 had surgical uterine evacuation, representing 22.0% (95% CI 18.8%, 25.5%). This percentage was strongly linked to the number of misoprostol doses, the length of dosing, the number of missed follow-up appointments, the publication date, the location, the method of dosing, the number of doses, and the time between dosing and assessment. A continuing pregnancy was present in 384 out of 6359 evaluable women (meta-analytic estimate 6.8%, 95% CI 5.3%, 8.5%). The number of women who were transfused or hospitalized due to an abortion was 26 out of 12,184 who were evaluable (meta-analytic estimate 0.7%, 95% CI 0.4%, 1.0%). A meta-analysis found that 78% of women were happy or very satisfied with their therapy, with a 95% confidence interval ranging from 71% to 85% among studies that reported patient satisfaction. They came to the conclusion that women seeking an abortion during the first trimester have a legitimate choice in misoprostol alone, as it is both effective and safe [24]. For the induction of a first-trimester abortion, vaginal misoprostol administration is more effective and more tolerated than oral administration after mifepristone administration [25].

## Conclusion:

Data shows that multigravida women predominated in both groups and their abortion histories were not substantially different. Post abortion hemoglobin levels showed a significant decrease in medical abortion cases compared to surgical abortion cases. Medical cases reported more frequent bleeding, cramps, nausea, and vomiting, while surgical cases had slightly higher infection rates. Despite the fact that analgesia is used more often in medical cases, both groups had good satisfaction rates.

## Figures and Tables

**Table 1:** Age distribution

**Age of Women (in years)**	**Medical**		**Surgical**		**P value**
	**Cases**	**%**	**Cases**	**%**	
< 20 yrs	6	8	5	6.7	0.9829
20-25 yrs	24	32	23	30.7	
25-30 yrs	28	37.3	29	38.7	
30-35 yrs	12	16	14	18.7	
> 35 yrs	5	6.7	4	5.3	
Total	75	100	75	100	

**Table 2:** BMI distribution

**Mean BMI (kg/m^2^)**	**Medical**		**Surgical**		**P value**
	**Cases**	**%**	**Cases**	**%**	
< 18.5	18	24	19	25.3	0.9843
18.5 - 22.99	13	17.3	12	16	
23-24.99	29	38.7	31	41.3	
25-29.99	12	16	11	14.7	
≥ 30	3	4	2	2.7	
Total	75	100	75	100	

**Table 3:** Mean gestational age

**Mean gestational age (weeks)**	**Medical**		**Surgical**		**P value**
	**Cases**	**%**	**Cases**	**%**	
08-Jul	14	18.7	10	13.3	0.5829
10-Sep	32	42.7	31	41.3	
12-Nov	29	38.7	34	45.3	
Total	75	100	75	100	

**Table 4:** Gravida distribution

**Gravida**	**Medical**		**Surgical**		**P value**
	**Cases**	**%**	**Cases**	**%**	
Primigravida	29	38.7	26	34.7	0.6112
Multigravida	46	61.3	49	65.3	
Total	75	100	75	100	

**Table 5:** History of abortion

**History of Abortion**	**Medical**		**Surgical**		**P value**
	**Cases**	**%**	**Cases**	**%**	
Yes	21	28	19	25.3	0.7119
No	54	72	56	74.7	
Total	75	100	75	100	

**Table 6:** Variable distribution

**Variables**	**Medical Abortion**
Misoprostol doses (mean ± SD)	5.03 ± 2.01
Induction-abortion time (h) (mean ± SD)	19.72 ± 7.11
Need for evacuation - cases (%)	9 (12%)

**Table 7:** HB level

**Hemoglobin level**	**Medical**		**Surgical**		**P-value**
	**Mean**	**SD**	**Mean**	**SD**	
Baseline level (g/dl)	11.7	± 2.4	11.9	± 2.1	0.5879
Level after abortion (g/dl)	10.6	± 1.9	11.1	± 2.5	0.2717
Mean change in Hb level (g/dl)	-1.1	± 0.6	-0.8	± 0.7	0.0054

**Table 8:** Reduction of HB

**Reduction in Hemoglobin level**	**Medical**		**Surgical**		**P value**
	**Cases**	**%**	**Cases**	**%**	
No reduction	1	1.3	10	13.3	< 0.001
0 - 1 g/dl reduction	43	57.3	56	74.7	
1 - 2 g/dl reduction	26	34.7	8	10.7	
> 2 g/dl reduction	5	6.7	1	1.3	
Total	75	100	75	100	

**Table 9:** Side effects

**Side effect within 14 days**	**Medical**		**Surgical**		**P value**
	**Cases**	**%**	**Cases**	**%**	
Bleeding	75	100	62	83	< 0.001
Cramping	44	59	25	33	< 0.001
Nausea	19	25	6	8	0.002
Vomiting	12	16	2	2.7	0.005
Fever	3	4	4	5.3	0.698

**Table 10:** Complication

**Complications**	**Medical**		**Surgical**		**P value**
	**Cases**	**%**	**Cases**	**%**	
Infection	0	0	2	2.7	-
Severe Bleeding	6	8	2	2.7	0.1462
Blood transfusion	0	0	1	1.3	-
Cervical trauma	0	0	0	0	-
Readmission after discharge	0	0	1	1.3	-
Analgesia need	42	56	14	19	< 0.001
Complications related to anesthesia	-		2	2.7	-

**Table 11:** Patient satisfaction

**Patient Satisfaction**	**Medical**		**Surgical**		**P value**
	**Cases**	**%**	**Cases**	**%**	
Women satisfied with method	71	94.67%	63	84.00%	0.0343
Women's preference to choose same method again, if required	45	60.00%	51	68.00%	0.3094

**Table 12:** Abortion method

**Abortion method**	**Cost in INR (Rs.)**
**Medical Abortion**	
Tab Mifepristone (single)	Rs. 400=00
Tab Misoprostol (Rs.20 x 4 tables)	Rs. 80=00
Total	Rs. 480=00
Surgical Abortion (D & E)	Rs. 1750=00
